# Dengue Virus Reporter Replicon is a Valuable Tool for Antiviral Drug Discovery and Analysis of Virus Replication Mechanisms

**DOI:** 10.3390/v8050122

**Published:** 2016-05-05

**Authors:** Fumihiro Kato, Takayuki Hishiki

**Affiliations:** 1 Department of Virology 1, National Institute of Infectious Diseases, Tokyo 162-8640, Japan; fumihiro@nih.go.jp; 2 Viral Infectious Diseases Project, Tokyo Metropolitan Institute of Medical Science, Tokyo 156-8506, Japan

**Keywords:** dengue virus, flavivirus, reporter replicon, replication, antiviral drug, high-throughput screening

## Abstract

Dengue, the most prevalent arthropod-borne viral disease, is caused by the dengue virus (DENV), a member of the *Flaviviridae* family, and is a considerable public health threat in over 100 countries, with 2.5 billion people living in high-risk areas. However, no specific antiviral drug or licensed vaccine currently targets DENV infection. The replicon system has all the factors needed for viral replication in cells. Since the development of replicon systems, transient and stable reporter replicons, as well as reporter viruses, have been used in the study of various virological aspects of DENV and in the identification of DENV inhibitors. In this review, we summarize the DENV reporter replicon system and its applications in high-throughput screening (HTS) for identification of anti-DENV inhibitors. We also describe the use of this system in elucidation of the mechanisms of virus replication and viral dynamics *in vivo* and *in vitro*.

## 1. Introduction

Dengue virus (DENV), which includes four serotypes (DENV1–4), is transmitted to humans by *Aedes* mosquitos and is the etiological agent of dengue fever and dengue hemorrhagic fever [1]. DENV causes an estimated 50–100 million cases of dengue fever, 500,000 cases of severe dengue (dengue hemorrhagic fever/dengue shock syndrome (DHF/DSS)), and more than 20,000 deaths each year in tropical and subtropical regions, representing a considerable public health threat in over 100 countries worldwide [2]. However, there are still no specific antiviral drugs or licensed vaccines against DENV infection.

DENV is an enveloped, positive-strand RNA virus belonging to the genus *Flavivirus* in the family *Flaviviridae*. Several other flaviviruses, including Japanese encephalitis virus (JEV), yellow fever virus (YFV), West Nile virus (WNV), tick-borne encephalitis virus (TBEV), and Zika virus (ZIKV), also are significant human pathogens [3,4]. The DENV genome consists of approximately 11 kb, containing one large open-reading frame (ORF). This viral RNA encodes a polyprotein that is processed by cellular and viral proteases into three structural proteins (capsid (C), pre-membrane (prM), and envelope (E)), which form the virus particle, and seven nonstructural (NS) proteins (NS1: essential for RNA replication, NS2A: inhibition of interferon signal, NS2B: cofactor of NS3 protease, NS3: protease and helicase activity, NS4A: induction of membrane rearrangements, NS4B: inhibition of interferon signal, and NS5: methyltransferase and RNA polymerase activity, inhibition of interferon signal). These NS proteins are responsible for replication of the viral genome but are not detectable in viral particles [4]. The ORF is flanked by highly structured 5′- and 3′-untranslated regions (UTRs), which play regulatory roles in translation of the viral proteins and viral RNA genome replication. In DENV and other flaviviruses, the presence of complementary sequences at the ends of the genome mediates long-range RNA-RNA interactions [5]. DENV RNA displays two pairs of complementary sequences (CS) required for genome circularization and viral replication [6,7]. The downstream 5′ CS pseudoknot (DCS-PK) elements enhance viral RNA replication by regulating cyclization [8].

The replicon system contains gene elements necessary for autonomous replication of the genome in cells. Expression of viral genes by replicon systems has been established in a number of positive-strand RNA viruses, such as Sindbis virus, poliovirus, Semliki Forest virus, human rhinovirus 14, coronavirus, and hepatitis C virus, and in various flaviviruses, including Kunjin virus, DENV, WNV, YFV, and TBEV [9,10,11,12,13,14,15,16,17,18,19]. In this review, we describe a replication-competent DENV subgenomic and full-length replicon system composed of reporter genes. This technology has improved dramatically in recent years and can be used for the screening of antiviral compounds and analysis of virus replication mechanisms.

## 2. DENV Life Cycle

**Figure 1:**
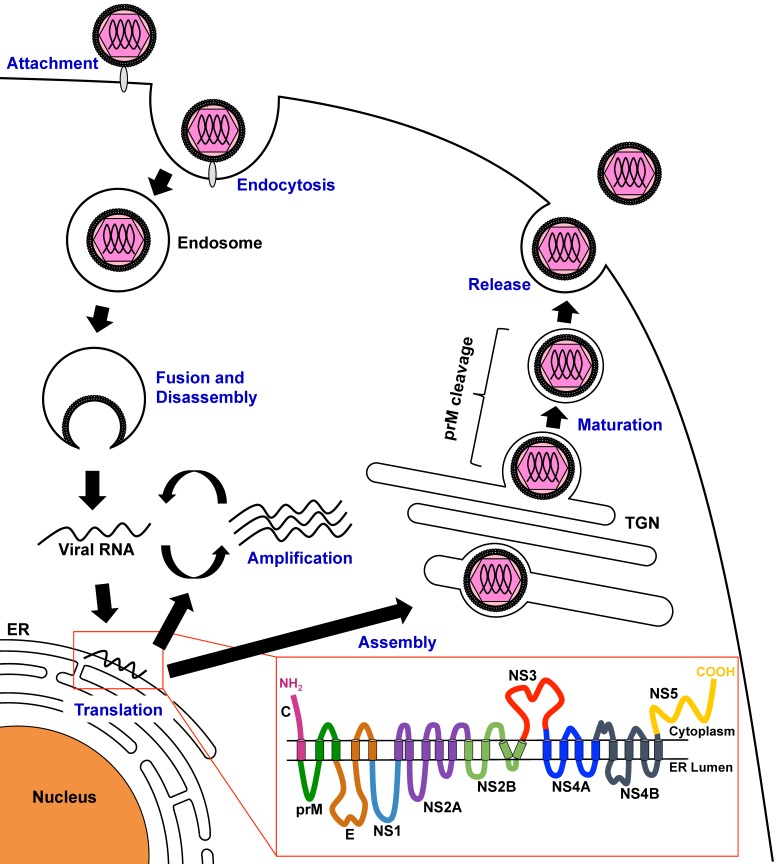
Schematic model of the dengue virus (DENV) life cycle. DENV particles bind to host cell factors and then enter the cell by clathrin-mediated endocytosis. After trafficking to endosomal compartments, envelope protein-mediated fusion of viral and cellular membranes occurs with changes in pH, allowing disassembly of the virus particles and release of single-stranded viral RNA into the cytoplasm, where translation occurs. The viral RNA is then translated to a polyprotein, which is processed by host cellular and viral proteases. Nonstructural (NS) proteins then replicate the viral RNA. Viral particle assembly occurs on the membrane of the endoplasmic reticulum (ER), and particles then bud into the ER as immature virus particles. During egress of the progeny virus particle through the secretory pathway, pre-membrane (prM) protein is cleaved by the cellular serine protease furin. Mature virus particles are released into the extracellular space. The red inset indicates the putative membrane topology of the viral proteins. TGN: trans-Golgi network.

**Figure 2:**
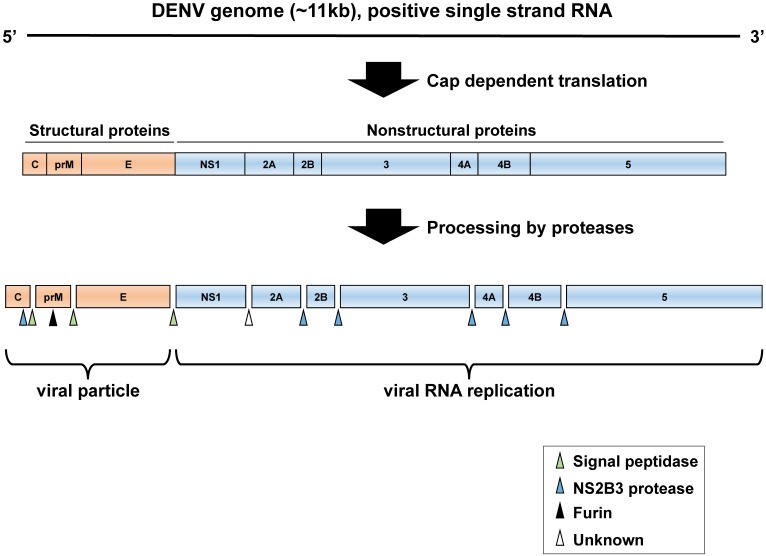
Schematic diagram of the DENV genome. The single-stranded viral RNA is translated by cap-dependent initiation scanning of the 5′- untranslated region (UTR). The translated polyprotein is processed by cellular and viral proteases into three structural proteins (capsid (C), pre-membrane (prM), and envelope (E) proteins) and seven NS proteins (NS1, NS2A, NS2B, NS3, NS4A, NS4B, and NS5). C, prM, and E proteins constitute the components of viral particles, whereas NS1–5 proteins function in the replication of RNA viral genome.

DENV attaches to cells via interactions between the E proteins of viral particles and cellular factors, including heparan sulfate, mannose receptor, dendritic cell (DC)-specific intercellular adhesion molecule 3-grabbing nonintegrin (DC-SIGN), and T-cell immunoglobulin and mucin domain (TIM) and Tyro3, Axl, and Mer (TAM) family proteins, on the target cell [20]. After binding, DENV is internalized into cells via clathrin-mediated endocytosis and traffic into the endosomal compartment, in which the low pH induces structural changes in the E protein, resulting in viral membrane fusion. The positive-stranded viral RNA is then released into the cytoplasm. The DENV genome is a single-stranded positive-sense RNA that functions as mRNA and is subsequently translated by the cell machinery, thus generating viral proteins in the endoplasmic reticulum (ER). DENV genome RNA replication is performed in a structure enclosed by a virus-induced intracellular membrane, called the replication complex (RC); the RC contains viral proteins, viral RNA, and host cell factors [21,22]. The assembly of DENV particles occurs in the ER, and the virions bud into the ER as immature virus particles that incorporate 60 trimeric spikes of the prM and E proteins. These immature virus particles are then transported through the trans-Golgi network (TGN). During egress, prM is cleaved by the cellular serine protease furin. Thereafter, infectious mature virus particles are released into the extracellular space (Figure 1 and Figure 2).

## 3. Subgenomic Reporter Replicon (Transient Expression of NS Proteins)

Subgenomic replicon systems including the coding region of NS proteins (NS1 to NS5) and the *cis*-acting element in the 5′- and 3′-UTR, which are needed for viral RNA translation and replication, are able to self-replicate in cultured cells. These replicon systems can be safely used to study many aspects of virus replication because of the lack of structural genes necessary for the production of virus particles. Consequently, subgenomic replicons are suitable for examination of viral genome replication independently of the process of viral particle assembly.

**Figure 3:**
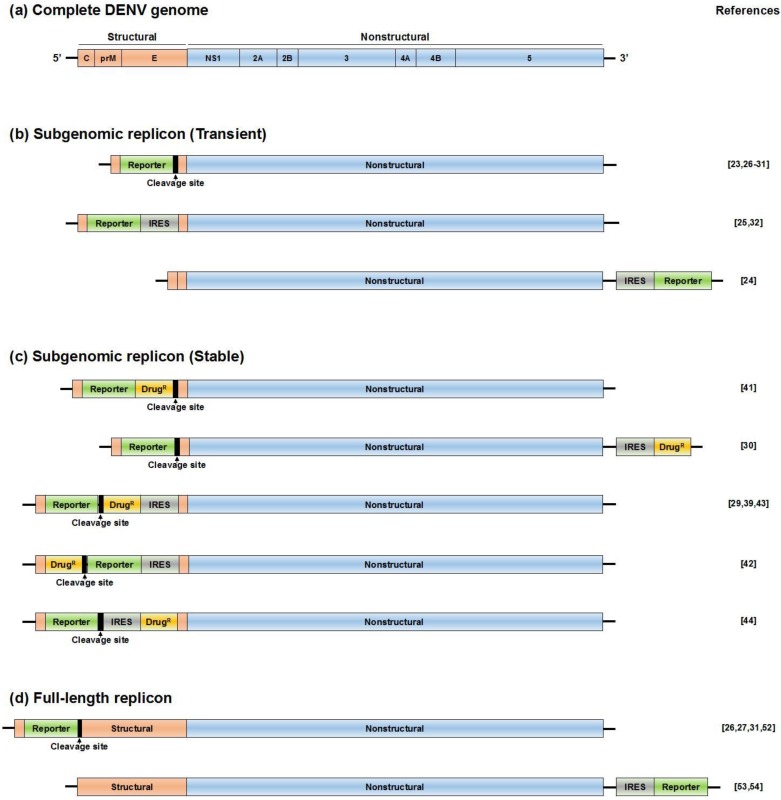
Schematic diagram of DENV reporter replicons. (**a**) Structure of the complete DENV genome; (**b**) subgenomic reporter replicons that are used for transient replication assays; (**c**) selectable subgenomic reporter replicons. The RNA supports stable expression of reporter and nonstructural (NS) proteins; (**d**) full-length reporter replicons that produce infectious viral particles. Reporter genes: *Renilla* (Rluc), firefly (Fluc), or *Gaussia* luciferase (Gluc) or green fluorescence protein (GFP); cleavage site: foot-and-mouth disease virus (FMDV) 2A or ubiquitin cleavage sequence; IRES: internal ribosome entry site; Drug^R^: drug-resistance gene.

The DENV replicon system described by Pang and colleagues does not contain a reporter gene for analysis of the level of DENV RNA replication [16]. To improve this system, researchers have developed new replicon systems (Figure 3b) [23,24,25,26,27,28,29,30,31,32]. Among them, DVRep, which harbors a firefly luciferase (Fluc) gene to replace the structural proteins [28]. The C-terminal 24 amino acids of the E protein, corresponding to the transmembrane (TM) domain, were included in the system to maintain the topology of the viral protein NS1 inside of the ER compartment. The luciferase was fused to the N-terminal 34 amino acids of the C protein, which contained the *cis*-acting element of 11 nucleotides complementary to the 3′ CS [33,34]. Furthermore, to ensure appropriate cleavage of luciferase from the viral polyprotein, foot-and-mouth disease virus (FMDV) 2A protease cleavage sites were introduced between the C-terminus of luciferase and the beginning of the TM domain of the E protein (Figure 3b, upper panel) [35]. After transfection of replicon RNA into BHK-21 and C6/36 cells, DVRep RNA translation and amplification could be monitored by measurement of luciferase activity. Moreover, an RNA element was identified in the 3′- UTR that differentially modulates viral replication in mosquito and mammalian cells in this replicon system.

Puig-Basagoiti *et al.* developed two types of subgenomic replicons [29]. One replicon contained a *Renilla* luciferase (Rluc) gene, which was substituted with the viral structure genes (DEN-1 Rluc-Rep). The other replicon was a FMDV 2A cleavage sequence inserted into the C-terminal of the luciferase of DEN-1 Rluc-Rep (DEN-1 Rluc2A-Rep). After transfection of DEN-1 Rluc-Rep RNA into BHK-21 cells, only a single luciferase peak was observed during the initial 10 h, and no further luciferase activity was detected up to 96 h. In contrast, after transfection of DEN-1 Rluc2A-Rep RNA into BHK-21 cells, two luciferase peaks were observed; the first peak was observed during the initial 10 h, and the second peak was observed after 10 h. These results suggested that the first luciferase peak may represent the translation of input RNA, whereas the second peak may represent viral RNA replication. Similar observations regarding the importance of the FMDV 2A sequence for cleavage between luciferase and the C-terminal fragment of the E protein were reported by Alvarez and co-workers [28].

Generally, a replicon is established by transfection of *in vitro* transcribed RNA. However, some reports have described DNA-based replicons, in which transcription is controlled by a cytomegalovirus (CMV) promoter prepared from Venezuelan equine encephalitis virus (*Alphavirus*), porcine reproductive and respiratory syndrome virus (*Arterivirus*), WNV, and DENV [36,37,38,39]. Leardkamolkarn *et al.* also successfully developed four types of subgenomic replicons, in which the GFP reporter gene was inserted into the structural region [31]. The DNA-based replicon is stable compared with RNA-based constructs, and the DNA-based replicon can be directly transfected (without *in vitro* transcription). Therefore, DNA-based replicons are simple and convenient to use for examination of the mechanism of DENV replication and for high-throughput screening (HTS) of anti-DENV compounds. Recently, we developed a DNA-based transient DENV-1 replicon encoding the Gluc reporter gene [32]. *Gaussia* is smaller than firefly or *Renilla* luciferase and generates a stronger signal [40]. Thus, as a major advantage of our system, *Gaussia* luciferase activity can be analyzed in culture medium, without the requirement for cell lysis.

## 4. Subgenomic Reporter Replicon Cells (Stable Expression of NS Proteins)

Stable viral replicating systems, called replicon cells, enable continuous viral RNA replication in cell culture through introduction of a drug-resistance gene into the viral genome. DENV replicon cells are useful tools for analysis of the replication mechanisms of the DENV genome; however, such cells are not suitable for HTS of anti-DENV agents. Therefore, researchers modified this system to introduce a reporter gene and to develop an appropriate assay system that could be used to analyze the amount of DENV RNA in cells by measuring reporter activity, such as luciferase or GFP [29,30,39,41,42,43,44].

The reporter within the DENV replicon contained Rluc ubiquitin, a selectable neomycin-resistance (*neo*) gene, and an encephalomyocarditis virus (EMCV) internal ribosome entry site (IRES) fragment; this was substituted for the viral structural genes to construct a replicon fragment that retained the 37 N-terminal amino acids of the C protein and 31 C-terminal amino acids of the E protein [29]. After transfection of the DENV replicon RNA into Vero cells, the replicon polyprotein driven by the DENV 5′-UTR and EMCV IRES was processed through cellular and viral protease-mediated cleavage, resulting in individual *Renilla* luciferase, neomycin phosphotransferase II conferring resistance to various aminoglycoside antibiotics, and NS proteins. The transfected cells were selected by geneticin treatment, allowing continuously replicating replicon cells to survive. Examination of the established cells demonstrated that viral proteins were expressed in all cells. Additionally, high levels of *Renilla* luciferase activity were maintained more than four months.

Some researchers have conducted studies in which the luciferase gene is replaced with the enhanced green fluorescent protein (EGFP) gene for construction of DENV replicon cells [41,42,44]. In this replicon assay system, readouts are easy to monitor by measurement of the fluorescence intensity of living cells. Indeed, Leardkamolkarn *et al.* carried out HTS and identified new anti-DENV compounds using this DENV replicon system.

## 5. Virus-like Particles

In recent years, single-round infectious virus-like particles (VLPs) that express reporter genes have been used widely as tools for studying several flaviviruses, including YFV and WNV [45,46]. VLPs are composed of an RNA reporter replicon genome that is packaged into virus particles by the viral structural proteins (C, prM, and E proteins) when provided in *trans*. The VLPs exhibit a structure similar to that of infectious live virus particles and can be used to study the entry and replication steps of the viral life cycle.

Qing *et al.* confirmed that DENV-VLPs are susceptible to the neutralizing antibody 4G2, which recognizes the fusion loop of domain II of the E protein, and to the anti-DENV compound NITD008, which is a nucleoside inhibitor of DENV RNA-dependent RNA polymerase (RdRp) [47,48,49]. Validation and optimization of VLPs for HTS of DENV inhibitors in a 384-well format yielded consistent, strong signals. Moreover, consistent with previous studies, they also found that the infectivity of VLPs was influenced by temperature [50]. Furthermore, Mattia *et al.* demonstrated the usefulness of VLPs for identification and measurement of neutralizing antibodies in human serum samples against all four DENV serotypes in large-scale, long-term studies [51].

## 6. Full-Length Reporter Replicon

The use of subgenomic replicon systems has improved our knowledge of the mechanisms of DENV replication; however, the genomic sequences used in these systems exhibit large deletions in the structural region (C, prM, and E coding sequences). Therefore, the subgenomic replicon cannot be used to examine several virus life cycle steps, such as entry, assembly, and release, or to identify antiviral agents targeting structural proteins. To overcome this limitation, researchers have developed full-length replicon systems [26,27,31,52,53,54]. Mondotte and colleagues first reported the development of a full-length reporter infectious virus, DV-R, and described the roles of two glycans in the E protein during DENV infection [53]. In this construct, the Rluc gene was introduced into the 3′-UTR of the DENV genome, with translation dependent on the EMCV IRES. After transfection of DV-R RNA into BHK-21 cells, the genome becomes self-replicative, resulting in production of infectious virus, although the DV-R RNA is longer than that of the parent strain, whereas growth curve analysis indicated that DV-R replication decreased in comparison with that of the parent strain; indeed, the titers at 24 and 48 h were significantly lower than those of the parent strain. Furthermore, DV-R infection is suppressed by heparin, an entry inhibitor, and replication abolished by an adenosine analog, also known as viral polymerase inhibitor. Therefore, DV-R provides a useful tool for investigating all steps of the virus life cycle.

Leardkamolkarn *et al.* generated a full-length (FL) replicon, FL-DENV/GFP, in which the GFP gene was inserted into the DENV genome between C and prM coding sequences [31]. However, infectious virus was not detectable in the cell culture supernatant, although GFP expression was detected in cells transfected with replicon RNA. Additionally, the amount of intracellular viral RNA was significantly decreased compared with that in the parent strain. These results suggested that GFP insertion in the viral RNA genome in this position affected the amplification of the virus genome and the infectivity of virus particles.

Reporter viruses are unstable; indeed, reporter genes are often deleted after a few rounds of viral replication [55]. To overcome this limitation, a DENV strain stably expressing luciferase, Luc-DENV, was developed. In this construct, the N-terminal 38 amino acids of the C protein were fused to the *Rluc* gene, and an FMDV 2A cleavage sequence was introduced into the 5′-UTR of DENV. After transfection of the Luc-DENV RNA transcript into BHK-21 cells, the viral titer from culture supernatants was lower than the parent virus [27]. To investigate the stability of Luc-DENV, researchers passaged the virus in Vero cells five times. As a result, luciferase activity was increased from the third to fifth rounds of passage compared with that during the first two rounds of passage virus. Furthermore, researchers showed that adaptive mutation in the NS4B gene could enhance viral RNA replication in a cell type-specific manner.

Schoggins *et al.* reported two types of full-length reporter replicons, which included luminescent or fluorescent reporters within the viral genome [52]. The first 25 amino acids of DENV C protein were repeated and introduced upstream of the reporter gene (Fluc or GFP), which was fused to a sequence encoding the FMDV 2A cleavage site. Both DENV constructs produced infectious viruses in culture supernatants through the *in vitro* transcribed RNA of the DENV reporter construct introduced into Vero cells. Interestingly, the infectious viral productivity of DENV-GFP was lower than that of the parent DENV strain. Furthermore, serial passage of the virus in the cell culture supernatant results in reduced GFP fluorescence, suggesting that the *GFP* gene was unstable during the DENV life cycle. Similarly, Zou *et al.* also reported that the Rluc incorporated into the full-length DENV genome was stable, whereas the GFP gene incorporated into the full-length DENV genome was unstable [27]. These results suggested that certain RNA elements within the GFP gene may interfere with DENV replication, thereby resulting in deletion of GFP during replication of GFP-DENV. Despite this limitation, the GFP-expressing replicon system still provides benefits for the study of DENV. For example, the replication level can be quantified by live-cell fluorescence imaging, a technique that provides rapid, simple screening. Furthermore, using this GFP-DENV replicon system, it is possible to use fluorescence-activated cell sorting (FACS) to measure and sort cells. Indeed, several antiviral effectors have been identified from an interferon-stimulated gene library using DENV-GFP.

For the DENV-Fluc, susceptibility to the well-characterized anti-DENV inhibitors mycophenolic acid (MPA), NITD008, and type I and III interferons is similar to that of parent DENV in Huh7 cells [49,56]. Interestingly, researchers further used this DENV-Fluc in an *in vivo* mouse model, together with substitution of a single amino acid mutation in NS4B, in order to examine the virulence of this mutation, which has been shown to enhance viral RNA synthesis in mice [57]. After infection of AG129 mice, which lack interferon-α, -β, and -γ receptors, bioluminescence imaging data showed that DENV localizes predominantly to lymphoid- and gut-associated tissues [58,59,60]. This observation is consistent with the results of another study on non-reporter DENV-infected AG129 mice. Furthermore, the use of DENV-Fluc has been used to demonstrate susceptibility to the anti-DENV compounds MPA and NITD008 and to neutralizing antibodies in AG129 mice. These results suggest that DENV-Fluc could provide a platform for screening and assessment of antiviral compounds and for analysis of DENV pathogenesis in living animals. 

## 7. Conclusions and Perspectives

The development of novel biological assays is required to continue advancements in the discovery of anti-DENV agents and to improve our understanding of the mechanism of DENV replication. Conventionally, anti-DENV activity is analyzed using infectious live virus. For example, quantification of the amount of infectious virus by plaque assay, observation of cytotoxic effects, or determination of viral RNA by reverse transcription polymerase chain reaction (RT-PCR). However, these assays are low throughput and cannot be used easily to screen large compound libraries. The development of the DENV replicon cell culture system is one of the most significant advances in DENV basic research and antiviral discovery. In recent years, exploitation of replication-competent reporter-expressing transient replicon systems, replicon cells, and single-round infectious particles has led to additional advancements in the field of DENV research. Furthermore, full-length reporter viruses are also useful tools for screening of inhibitors that affect all steps of the DENV life cycle and for examination of the mechanism of DENV replication and pathogenesis *in vivo* and *in vitro*. These model systems will further expand our understanding of virus-host interactions, viral pathogenesis, and immunological responses to DENV infection, thereby facilitating the development of drugs and vaccines.
